# Construction of cDNA library of *Dalbergia odorifera* induced by low temperature stress and screening of low temperature tolerant genes

**DOI:** 10.1371/journal.pone.0318935

**Published:** 2025-04-01

**Authors:** Shaocui Li, Xia An, Fayong Li, Yining Chen, Xiaowen Li

**Affiliations:** 1 Zhejiang Xiaoshan Institute of Cotton & Bast Fiber Crops, Zhejiang Institute of Landscape Plants and Flowers, Zhejiang Academy of Agricultural Sciences, Hangzhou, China; 2 Wenzhou Key Laboratory of Resource Plant Innovation and Utilization, Zhejiang Institute of Subtropical-Crops, Zhejiang Academy of Agricultural Sciences, Wenzhou, China; Nuclear Science and Technology Research Institute, IRAN, ISLAMIC REPUBLIC OF

## Abstract

To systematically analyze the gene function of *Dalbergia odorifera*, the seedlings of *D. odorifera* were treated with low-temperature stress for 6 h. Total RNA was extracted from a mixture of seedling roots, stems, and leaves, and a low-temperature-induced *D. odorifera* yeast cDNA expression library was constructed. The library volume was 1.032 × 10^8^ CFU, and the PCR (Polymerase Chain Reaction) identification of the library bacterial fluid showed that the amplification was around 1000 bp, with a single randomly distributed band, indicating that the library had been recombinantly inserted into the pYES2 vector. The GO (Gene Ontology) analysis showed that the library genes were mainly involved in metabolic and stress signaling pathways. The KEGG (Kyoto Encyclopedia of Genes and Genomes) pathway enrichment analysis showed that the genes were primarily related to energy and metabolic pathways. Twenty-one genes were screened or obtained at -20°C for low-temperature tolerance. In addition, the organ expression profiles of the candidate genes were analyzed based on RNA-seq data, and the expression profiles of the candidate genes under low-temperature stress were also examined. The construction of the yeast library provides genetic resources for the analysis of the mechanism of low-temperature tolerance of *D. odorifera*, which is important for comprehending and utilizing the genetic resources of *D. odorifera*.

## Introduction

Abiotic stress refers to any abiotic factor that adversely affects a plant in a given environment, resulting in a range of responses from changes affecting biological processes (e.g., gene expression and cellular metabolism) to growth and development [1,2]. Abiotic stresses mainly include temperature stress, drought stress, flood stress, salinity stress, metal stress and nutrient stress. Among them, low temperature stress is one of the most serious environmental stresses affecting higher plants [3,4]. It was shown that low-temperature stress not only affects plant growth and development, but also produces significant differences in the geographical distribution of plants [5,6].

Low temperature stress is categorized into cold damage (<0°C) and freezing damage (>0°C), and the damage is demonstrated by disrupting cell membranes, affecting protein and enzyme activities, and affecting photosynthesis. In plants, low temperature stress induces different response pathways [7]. The various response pathways may also be associated in some way. In recent years, low-temperature responsive genes and their regulatory networks have been recognized and reported. Among them, ICE-CBF-COR is a low-temperature-induced regulatory pathway in plants and is one of the most widely reported pathways. Low temperature induces the ICE-CBF-COR pathway to induce expression, which is followed by the activation of expression of appropriate downstream genes encoding osmotic-regulatory substances [8]. ICE is a MYC-type basic helix-loop-helix family transcription factor (TF) that induces the expression of CBF (core binding factor), including the AP2 structural domain protein [9]. CBF, also known as dehydration-responsive element-binding proteins (DREBs), is a member of the APETALA2/ethylene-responsive factor (AP2/ERF) family and plays an important role in cold adaptation [10,11]. The AP2/ERF family is one of the largest TF families in plants and is characterized by at least one AP2 DNA-binding structural domain [12]. Subsequently, CBF regulates cold-responsive (COR) gene expression by binding to a cis-element in the COR gene promoter (CRT: TGGCCCGAC) [13,14].

The ICE1 (inducer of CBF expression 1) was the first upstream CBF regulator to be identified [15]. It specifically binds to the MYC (Myelocytomatosis) cis-element (CANNTG) in the CBF1-3 gene promoter and induces gene expression at low temperatures [16]. ICE transcription factors have been identified to regulate the response to abiotic stresses in numerous plants. Overexpression of the stress-responsive gene *DlICE1* in *Dimocarpus longan* enhances cold tolerance in transgenic *Arabidopsis thaliana* [17]. *ICE2* expression was induced by cold in cotton, and overexpression of *GthICE2* in *A. thaliana* resulted in the observation of lower MDA (Malondialdehyde) content and higher SOD (Superoxide Dismutase) and POD (Peroxidase) activities [18]. In addition, in strawberry (*Fragaria vesca*), *FvICE1* directly binds to the CRT/DRE elements in the promoters of CBFs to positively regulate the expression of *FvCBF1*, *FvCBF2*, and *FvCBF3*, which in turn regulates the expression of *FvCOR413* and *FvKIN1*, resulting in the enhancement of strawberry’s ability to survive under cold and drought stress [19]. In banana (*Musa acuminata*), *MaDREB1F* was also found to co-trigger the accumulation of soluble sugars and proline, activate the antioxidant system, and promote the synthesis of jasmonate and ethylene, which confers drought and cold resistance to the plant [20]. In conclusion, the series of chain reactions induced by abiotic stresses is a multilevel, multiprocess molecular mechanism involving perception, signal transduction, transcription, processing, protein translation and modification, as well as a complex response mechanism with multiple genes, signaling pathways and metabolic processes [21].

*Dalbergia odorifera* T. Chen, belongs to the Fabaceae family, and is endemic to Hainan, China. It has been widely introduced into Guangxi, Guangdong, Fujian and Yunnan in China [22,23]. It is known to be rich in secondary plant metabolites such as terpenes and flavonoids and is consequently used in the treatment of various diseases, such as analgesic, angiogenesis and anticancer activities [24,25]. In addition, *D. odorifera* has been regarded as a valuable plant for manufacturing pricey furniture and exquisite crafts [26]. Due to its high medicinal and commercial value, *D. odorifera* has extensive exploitation. However, *D. odorifera* is rapidly disappearing: only a limited number of trees are found in parts of their original habitat, as highly fragmented populations are now remaining in the forests of Hainan Island (China) [27]. According to reports, the expression of these metabolites used for disease treatment in *D. odorifera* is induced by various abiotic or biotic stresses [28]. Indeed, soil water availability, temperature, light regime, or nutrient supply are deeply involved in plant metabolites [29]. Medicinal plants under water deficiency conditions showed considerably higher concentrations of natural products related to secondary metabolites than identical plants of the same species under ordinary environments. Those natural products include simple or complex phenols and numerous terpenes, alkaloids, flavonoids, or glucosinolates [30]. To defend against abiotic stress, plants have evolved various defense mechanisms, such as changes in growth habits, morphological, physiological, and biochemical [31]. At present, there is also much evidence that the synthesis of secondary metabolites in plants subjected to abiotic stresses may be part of the defense mechanism [32]. Various secondary metabolites help improve stress resistance and respond to the surge of reactive oxygen species (ROS) or regulating cell wall structure [28].The negative impact of global climate change on forest tree species is increasing yearly. Low-temperature stresses occur frequently and are very damaging to plants, which can affect crop yield and quality. Therefore, the response to abiotic stresses has also become an important research element. Results showed that in *D. odorifera* seedlings, exogenous MT (Melatonin) and Ca^2+^ application improved growth traits and relieved injuries, whereas chilling stress caused injuries and reduced growth [33]. The RNA-Seq analyses pointed out that cold acclimation already fixed the different gene expression patterns of *D. odorifera* [34]. Understanding the molecular mechanism of plant response to low-temperature stress can provide a reference for improving genetic improvement and molecular breeding for low-temperature tolerance in plants.

Traditional methods for confirming apparent gene function depend on the generation of loss-of-function and gain-of-function mutational resources, but have intrinsic shortcomings. The building of mature mRNA full-length cDNA libraries promotes the characterization of gene function and the manipulation of gene expression in exogenous systems by generating tagged versions of natural proteins [35]. A series of candidate genes involved in Cadmium (Cd) stress response were successfully screened and characterized using a cDNA library of domesticated *Sedum plumbizincicola* using a cadmium -sensitive yeast mutant (Δ*ycf1*) [36].

In the present study, we constructed a cDNA library of *Dalbergia odorifera*, from which we identified 22 genes associated with low-temperature tolerance. Our work attempts to provide a rational and effective tool for bulk digging of functional genes related to low-temperature response and to provide insights into the mechanism of low-temperature high tolerance. Specifically, these identified genes hold significant potential for use in the targeted selection and breeding of transgenic plants capable of thriving under low-temperature conditions.

## Materials and methods

### Plant materials and cold stress treatment for cDNA library construction

For this experimental, *D. odorifera* plants were provided by the Zhejiang Institute of Subtropical Crops (Wenzhou, Zhejiang). The average temperature in Wenzhou ranged from 18.78°C to 19.38°C, while the lowest temperature ranged from 1°C to - 4.3°C. In the greenhouse, the temperature was above 10°C in winter. All plants were then put in the greenhouse, which acted as the temperature control. The initial temperature of the greenhouse was set at 10°C on the first day, and then was gradually decreased to -3°C [34]. The seedlings grow in a 30 centimeter diameter pot filled with red soil, sand, and coconut fiber (2:2:1, v/v/v) [37].Plants with similar growth conditions were then all placed in a greenhouse standardize temperature conditions. The subjects of this study were four-year-old *D. odorifera* seedlings grown in pots, selected for their uniformity in growth, with a height of approximately 1.5 m and a stem diameter at ground level of around 4 cm. In order to extract the RNA of *D. odorifera* under low-temperature stress and construct a full-length cDNA library, healthy rooted seedlings treated at -3°C for 60 min and were collected, and three tissues (roots, stems, and leaves) were collected separately and immediately frozen in liquid nitrogen to extract the RNA.

### RNA extraction and library construction

TotalRNA isolation was extracted by the Trizol (Life Technologies, Carlsbad, CA, USA) method according to the manufacturer’s instructions. The quality of all RNA samples was determined by 1% agarose gel electrophoresis and detection of the A260/280 and A260/A230 by NanoDrop2000 spectrophotometer (Thermo Scientific, Wilmington, DE, USA). Oligo (dT) magnetic beads were then used to purify the extracted mRNA.

The total RNA of *D. odorifera* was used as a template for the reverse transcription synthesis of the first-strand cDNA. Subsequently, the synthesized first-strand cDNA was used as a template to synthesize the second-strand cDNA under the catalytic action of DNA Ligase (10 U μL^-1^), DNA Polymerase Ⅰ (10 U μL^-1^), RNase H (2 U μL^-1^) and T4 DNA Polymerase. The all primer sequences are shown in S1 Table.

A triple-frame recombination junction was attached to the obtained second strand cDNA, followed by cDNA hierarchical isolation and collection. The collected cDNAs were subjected to recombination reaction with the pYES2.0, and the reaction products were transformed into *E. coli* cells. After the transformation stage, the cells were transferred to SOC medium and cultivated at 37°C for about 60 min. Subsequently, the bacterial fluid was divided into two parts, and one part of the stock solution was taken out for subsequent determination of library capacity, recombination rate and insert fragment length. The remaining stock solution was added with glycerol to a final concentration of 20% and stored at -80°C, which is the library liquid.

### Yeast transformation, and annotation of the cDNA library

Yeast library plasmids were extracted using the High Purity Plasmid Macro Extraction Kit (Tiangen, DP116), and the plasmids extracted from the cDNA library were transformed into the yeast strain BY4741 using the lithium acetate method. Take 100 μl of stock solution and dilute 10^-1^, 10^-2^, 10^-3^ respectively and spread on SD-U solid medium. Yeast library titer, library capacity, and next-generation sequencing (NGS) assay were performed. The remaining liquid was spread in 150 mm SD-U plates and incubated at 28°C for 3-4 d. After the clones grew, 24 clones were randomly picked for PCR validation to check the library quality.

### Screening of the cDNA library

For further screening of low-temperature tolerance genes in *D. odorifera*, the above bacteria were activated and coated on SG-U solid medium. Meanwhile, yeast cells containing an empty vector (*Δ*ycf1_EV) were used as a negative control for preliminary screening to determine the screening conditions. For low-temperature treatment, transformed cDNA libraries and control yeast fluids were cultured with liquid medium and treated at -20°C for 72h.

### GO and KEGG enrichment analysis

The clean sequences obtained after removing poor quality and mistranslated sequences from the data were mapped to the *D. odorifera* reference genome and the corresponding genes were detected in the genome database. The GO terms of the isolated genes were extracted (http://www.geneontology.org). The KEGG pathway (http://www.kegg.jp/kegg/kegg1.html) of the isolated gene was retrieved and visualized.

### Expression pattern detected by transcriptome and genomics data

We extracted RNA-seq data of low-temperature stress from previous studies to investigate the expression of candidate genes under low-temperature stress (PRJNA1148092) [34]. The genomics data of low-temperature stress was obtained from GIGADB DATASETS (http://gigadb.org/dataset/100760#). The FPKM (fragments of transcripts per kilobase per million mapped reads) values of the genes in different tissues and under low-temperature treatments (low temperature, rewarming) are shown in S2-S3 Table. Expression levels of the candidate genes were visualized and analyzed using TBtools. Heatmaps show tissue-specific expression profiles and expression under low temperature stress.

### Quantitative reverse transcription-PCR (qRT-PCR) expression analysis

Total RNA was isolated and converted into cDNA using TRIzol Reagent (Invitrogen, Waltham, MA, USA) and PrimeScript™ RT Master Mix (TaKaRa), respectively. The transcript levels were quantified using SYBR Green Premix Ex Taq II (TaKaRa) on a 7300 Real-Time PCR (Applied Biosystems, Carlsbad, CA, USA). Three independent experiments were performed, with three biological replicates per treatment. To normalize gene expression levels, the 2^-ΔΔCt^ method was calculated.

## Results

### Extraction of total RNA from different treatments of *D. odorifera*

**Fig 1:**
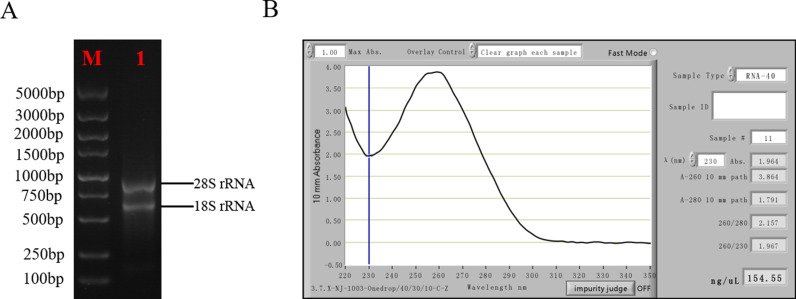
RNA isolation of *D. odorifera.* (A) Results of RNA electrophoresis assay, M: Marker, 1: total RNA of *D. odorifera* samples. (B) Results of RNA measurement by spectrophotometer.

Collection of samples treated at low temperature (-3°C) for 60 min was performed for total RNA extraction. To characterize whether the extracted RNA could be used for downstream library construction, 2 μl was taken up for agarose electrophoresis detection and spectrophotometric measurement, respectively. The quality of the total RNA samples is shown in Fig 1, which clearly shows the bands corresponding to the complete 28S and 18S rRNA. The A260/A280 ratio of total RNA was 2.157, A260/A230 ratio was 1.967, and the concentration was 154.55 ng/μL, indicating that the isolated and purified mRNA did not decompose and had good quality, which could be used as the initial sample of the library.

### mRNA purification, reverse transcription, and obtaining ds cDNAs

**Fig 2:**
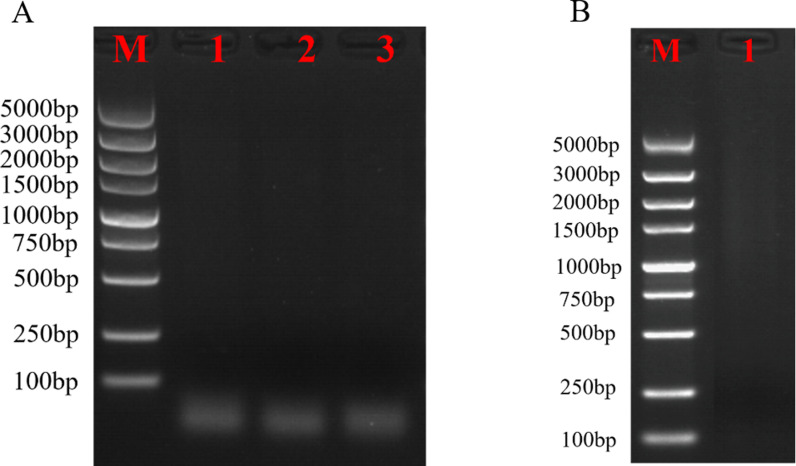
The synthesis and normalization of cDNA. (A) The synthesis of the double-strand cDNA. M: Marker, 1: P1-F/P4-R amplified ds cDNA; 2: P2-F/P4-R amplified ds cDNA; 3: P3-F/P4-R amplified ds cDNA; (B) the normalization of the ds cDNA. M: Marker, 1: purification results of three ds cDNA mixes.

The mRNA was purified and reverse transcribed into single-stranded cDNA using the magnetic bead method, followed by the use of double-stranded cDNA synthesized by LD-PCR. The detection of 5 μl product was carried out by agarose electrophoresis, and the results showed that the ds cDNA showed diffuse bands, indicating that the quality of the synthesized cDNA was better, and the bands of all sizes were present (Fig 2). As shown in Fig 2B, ds cDNA was purified and homogenized using magnetic beads. Pipetting 5 μl from the purified solution for agarose electrophoresis detection, the ds cDNA showed diffuse bands and no obvious bright bands, indicating successful homogenization. Below 500 bp, almost no bands were detected, indicating that the small fragments were removed cleanly (Fig 2B).

### The cDNA library was successfully constructed with a large library capacity and appropriate inserted fragments

**Fig 3:**
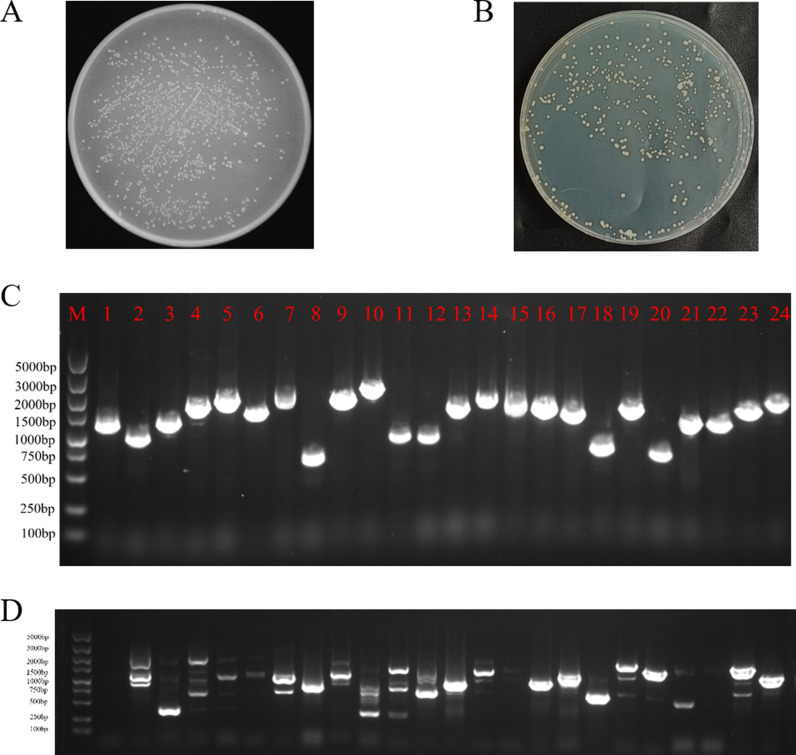
The quantification of the library by sequencing of positive colonies and plate counting. M: DL 2000 DNA Marker; 1–24: PCR products of 24 colonies. (A) *E. coli* colony count; B. plate counting of 10^-5^-fold diluted yeast cells from the yeast library; (C, D) Agarose gel electrophoresis of PCR products from 24 randomly selected *E. coli* and yeast colonies.

The cDNA was ligated into the pYES2.0 vector using homologous recombination. The recombinant vector was transfected into *E. coli* TOP 10 receptor cells by electro transformation. In order to determine the library capacity, 10 μl of the bacterial broth from the incubation of electro transformation was aspirated and diluted 10,000 times, from which 100 μl was coated in solid medium containing ampicillin and incubated inverted at 37°C overnight. The transformed bacteria were diluted for plate counting and the result showed that the bacteria library was 1.032 × 10^8^ CFU/ mL and the total number of clones was 2.064 ×  10^8^ (Fig 3A).

In order to detect the distribution of bands in the library, 24 clones were randomly selected from the plate for colony PCR and agarose gel electrophoresis detection, and it can be shown that all the bands appeared around 1000 bp, indicating that the average size of the library fragments was 1000 bp (Fig 3C).

The library plasmid was then transformed into yeast BY4741 to obtain the yeast library, and the diluted yeast cells were grown on the YPDA plates. The counting result showed that the yeast library capacity was 4.8 × 10^8^ CFU/mL ×  1 m L =  4.8 × 10^8^ CFU/μg of cDNA insert (Fig 3B). In addition, yeast colony PCR identification showed that the recombination efficiency of the amplified library also met the standard for constructing high-quality libraries (Fig 3D).

### GO functional classification and KEGG metabolic pathway analysis

**Fig 4:**
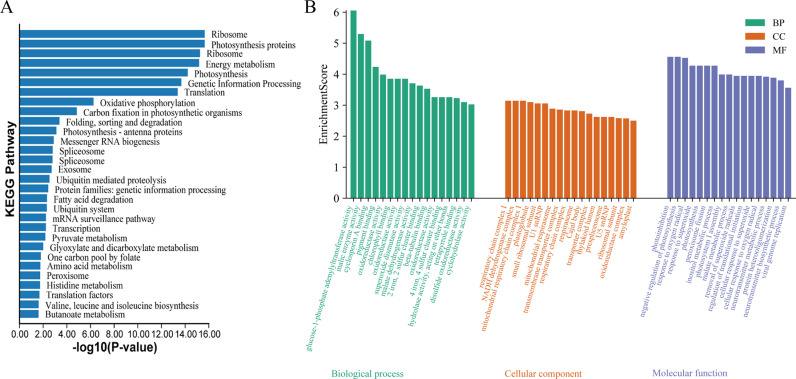
KEGG and GO analysis of cDNA library genes. (A) KEGG analysis; (B) GO analysis.

All positive clones of yeast screening library plates were collected and the cDNA libraries were subjected to NGS sequencing after PCR. Subsequently, the sequencing data were compared with the genome. To better understand the biological functions of the genes in the cDNA library of *D. odorifera* yeast, we performed KEGG analysis. KEGG annotation showed that the yeast library genes were significantly enriched in ribosome, photosynthesis proteins, energy metabolism, photosynthesis, genetic information processing and translation pathways. This result suggests that these pathways play an important role in the molecular mechanisms of low-temperature tolerance in *D. odorifera*. Further, the GO function classification of the genes in the library was performed to provide the annotation of biological process (BP), molecular function (MF), and cellular component (CC). As shown in Fig 4, photoinhibition, negative regulation of photosynthesis, and response to oxygen radical were the top three GO items for molecular function. In CC, respiratory chain complex I, NADH dehydrogenase complex, and mitochondrial respiratory chain complex I ranked the top three respectively; in biological process, glucose-1-phosphate adenylyl transferase activity, malic enzyme activity, and cyclosporin A binding were the three most important GO items.

### The low-temperature tolerance genes of *D. odorifera* screened from yeast cDNA library

**Fig 5:**
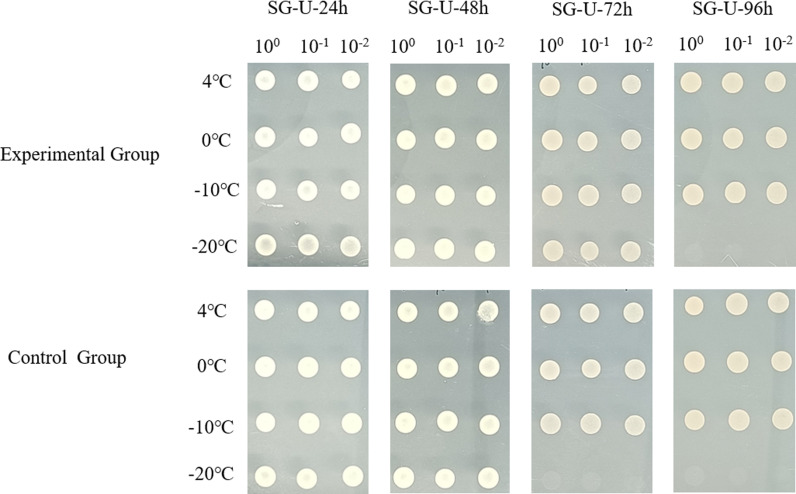
The growth trend of transgenic yeast BY4741 at different temperatures. Different temperatures represent the cultivation temperature of corresponding clones.

**Fig 6:**
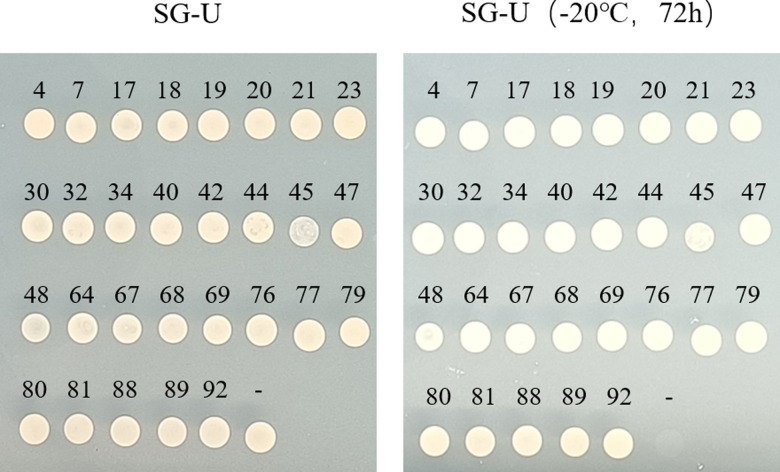
Screening of a cDNA library from *D. odorifera.* The growth state of randomly selected transgenic yeast monoclonals under -20 °C. The figures on the image represent different clone ID numbers.

In order to further explore more genes for low-temperature tolerance in plants, a cDNA library of *D. odorifera* was utilized to identify low-temperature tolerance genes by screening test. The cDNA library and empty pYES2 were transformed into yeast (BY4741) and cultured at different temperatures. The results showed that the control group (empty pYES2.0) could not grow at -20°C. While the experimental group (cDNA library) could, so we chose -20°C as the library screening condition (Fig 5). Ninety-six independent clones were randomly selected to observe their growth at low temperature. After revalidation, only 29 asexual lines were all able to grow at -20°C. The negative control could not grow, indicating that the yeast clones screened at -20°C had greater low-temperature tolerance than the negative control yeast (Fig 6). All low-temperature-tolerant clones were collected and sequenced (S4 Table).

### Functional annotations of the genes for low-temperature tolerance in *D. odorifera*

A total of 22 candidate proteins were obtained by analyzing the sequencing results. The NCBI database (https://ncbi.nlm.nih.gov/) was used to predict the function of these 22 candidate proteins (Table 1). Among the identified genes, 9 were categorized as proteases (*DoCYP450*, *DoGLY*, *DoVPE*, *DoPAO*, *DoTPA*, *DoCoxE*, *DoVHA*, *DoPsaA*, *DoBPL*), 7 as stress-responsive (*DoLTTP1*, *DoARM*, *DoLTTP2*, *DoRBD*, *DoLTTP3*, *DoFBA*), and 2 as protein degradation-related genes (*DoDCN*, *DoWD40*). Additionally, single instances were observed for light-responsive genes, genes involved in the transcriptional regulation of macromolecular biosynthesis, and kinase-related proteins. Notably, 4 novel genes emerged from this analysis, which designated as *DoLTTP* (Low Temperature Tolerance Protein) genes. These newly discovered genes appear to be unique to *D. odorifera* and may play pivotal roles in its low temperature tolerance mechanisms. The nucleotide sequences of all genes are shown in S5 Table.

**Table 1:** The functions of 22 proteins were predicted using the NCBI.

gene name	gene ID	Category	annotation	CDS homologues	reference
DoLTTP1	evm.model.scaffold_35.700	stress response	stress response	uncharacterized protein	
DoARM	evm.model.scaffold_227.20	stress response	abiotic stress and plant development	Armadillo/beta-catenin-like repeat	[38]
DoDCN	evm.model.scaffold_241.143	protein degradation	ubiquitin ligase, redox sensor, and regulation of gene expression	DCN1-like protein 2	[39]
DoLTTP2	evm.model.scaffold_100.484	stress response	stress response	uncharacterized protein	
DoELF1	evm.model.scaffold_5.1582	regulation of transcription	responsible for tillering, growth of seminal roots, and salt stress	Transcription elongation factor Elf1	[40]
DoRBD	evm.model.scaffold_5.22	stress response	stress response	S1 RNA binding domain	[41]
DoCYP450	evm.model.scaffold_100.231	protease	Cellular oxidase	Cytochrome P450	[42]
DoGLY	evm.model.scaffold_28.403	protease	detoxification	Glyoxalase/Bleomycin resistance protein/Dioxygenase superfamily	[43]
DoVPE	evm.model.scaffold_369.100	protease	defense responses and signaling	vacuolar processing enzyme	[44]
DoPAO	evm.model.scaffold_69.408	protease	Induction and catalysis	Pheophorbide a oxygenase	[45]
DoTPA	evm.model.scaffold_39.658	protease	stress response	thiol protease aleurain-like isoform X1	[46]
DoCoxE	evm.model.scaffold_28.461	protease	unknown	VWA domain containing CoxE-like protein	[47]
DoVHA	evm.model.scaffold_111.278	protease	catalyze	V-type proton ATPase subunit e1	[48]
DoLTTP3	evm.model.scaffold_307.277	stress response	stress response	uncharacterized protein	
DoPsaA	evm.model.scaffold_22.3	protease	light-induced regulatory	Photosystem I psaA/psaB protein	[49]
DoGPAT9	evm.model.scaffold_39.426	macromolecule biosynthetic	glycerol-3-phosphate acyltransferase 9	glycerol-3-phosphate acyltransferase 3-like	[50]
DoLTTP4	evm.model.scaffold_376.301	stress response	stress response	uncharacterized protein	
DoBPL	evm.model.scaffold_142.95	protease	protein ligase 2 isoform X1	Biotin protein ligase C terminal domain	[51]
DoNdr	evm.model.scaffold_394.364	kinase	kinase	Ndr family	[52]
DoLir1	evm.model.scaffold_233.220	light response	light response	Light regulated protein Lir1	[53]
DoWD40	evm.model.scaffold_12.86	protein degradation	WD40-like family	WD40-like family	[54]
DoFBA	evm.model.scaffold_222.1005	stress response	fructose-bisphosphate aldolase 8, cytosolic	Fructose-bisphosphate aldolase class-I	[55]

### Expression of candidate genes in different organs

**Fig 7:**
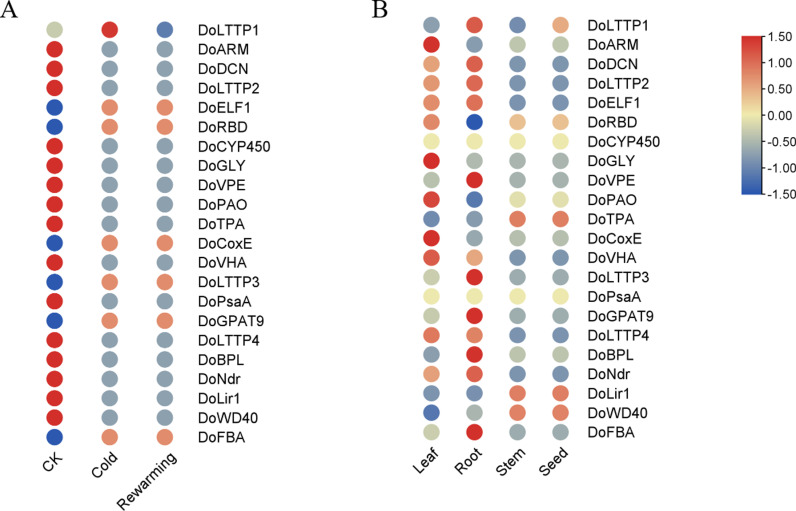
Expression patterns of low-temperature-responsive genes. A. Heatmap of gene expression patterns at different temperatures; B. Heatmap of gene expression patterns in different tissues. CK: The initial temperature of the greenhouse was set at 10 °C. Three biological replicates were analyzed. Red and blue respectively indicate negative and positive Z-scores for the gene expression levels.

The tissue expression profiles of the 22 genes are shown in Fig 7. In seed and stem, highly expressed genes were associated with stress response related *DoTPA*, light response DoLir1, and protein degradation DoWD40; 5 genes highly expressed in root were associated only with proteases (*DoVPE*, *DoBPL*), stress response related (*DoLTTP3*, *DoFBA*), macromolecule biosynthetic (*DoGPAT9*). 7 genes highly expressed only in leaves were associated with stress response (*DoARM*, *DoRBD*, *DoLTTP4*), proteases (*DoGLY*, *DoCoxE*, and *DoVHA*), and kinases (*DoNdr*). The highest number of highly expressed genes was found in leaves.

**Fig 8:**
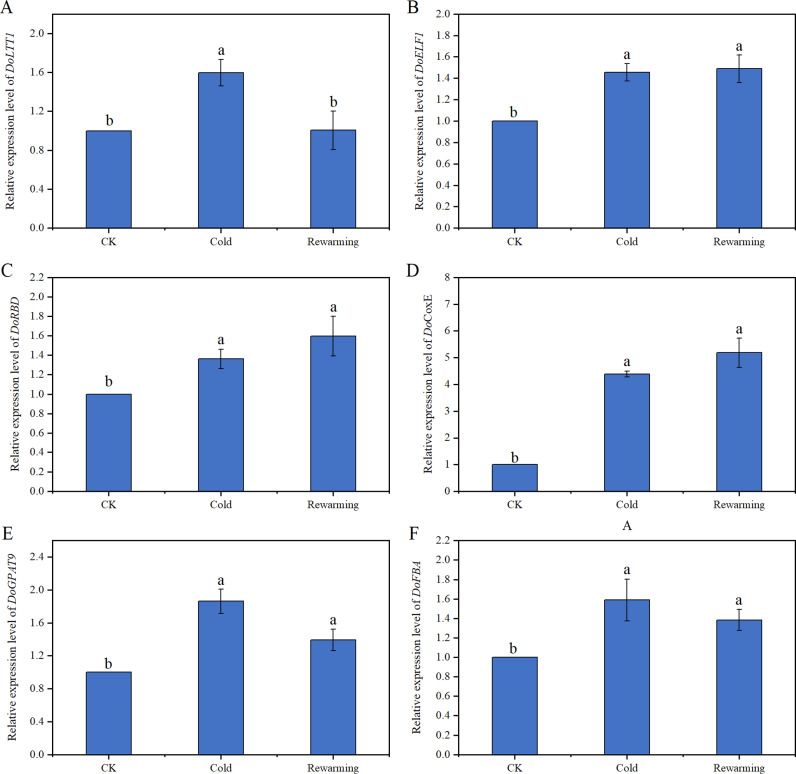
qRT-PCR analysis of the expression profiles of six candidate genes. Data are presented as the mean ± SD of three independent replicates. Different letters above bars indicate significant differences among treatments (n = 3, *p* <  0.05).

To determine the genes involved in cold stress response, we obtained the expression profiles of six selected genes upon exposure of the plants to a low temperature (-3°C) through qRT-PCR analysis (Fig 8). Under cold treatment, the expression levels of three genes (*DoLTT1*, *DoGPAT9*, and *DoFBA*) were rapidly upregulated; while the expression of *DoELF1*, *DoRBD*, and *DoCoxE* increased at cold and rewarming treatment (Fig 8).

## Discussions

The construction of a cDNA library of low-temperature-tolerant yeast in *D. odorifera* can efficiently screen low-temperature-tolerant genes, which is of great significance for understanding how plants adapt to low-temperature environments. As an endangered species, the conservation and rational utilization of its genetic resources are particularly important. The construction of a cDNA library will not only help to protect its genetic diversity, but also provide a scientific basis for the rational development and utilization of the biological resources of *D. odorifera*.

Although there are multiple methods for screening low-temperature tolerance genes, screening target genes by constructing cDNA libraries is still a fast and effective method. Yeast, as a model organism with a clear genetic background and a short growth cycle, can be screened for target genes in a short time, even if the genes are isolated from non-model plants, such as *D. odorifera*. Library capacity is an important indicator of the quality of a cDNA library, and a quality cDNA library usually contains at least 5 × 10^6^ clones [56]. The library capacity of our library was 1.032 × 10^8^ clones, which meets the requirement of high-quality library capacity. In this study, 24 randomly selected colonies were detected by PCR, and the length of the insert fragments was around 1000 bp. The proportion of long fragments in the library was large, indicating that the integrity of the insert fragments was good, which was suitable for sequencing and gene screening in the future.

The role of GO database is to provide a standardized framework for biological research to describe the functional properties of genes and gene products (such as proteins). The GO classification can help us to understand the biological significance of the genes. The results of GO analysis showed that the cDNA library of *D. odorifera* cDNA library was rich in gene functions, and the genes expressed in low-temperature-resistant genes mainly included three groups: cellular components, molecular functions, and biological processes. Among the cellular components, respiratory chain complex I accounted for the highest proportion; among the molecular functions, photoinhibition accounted for the highest proportion; and among the biological processes, glucose-1-phosphate adenylyl transferase activity was the most prominent. Among KEGGs, ribosome, photosynthesis proteins, and energy metabolism were the most prominent. At the same time, they are also metabolic processes with important roles in low-temperature response.

In addition, with functional annotation we also found that evm. model. scaffold_222.1005 was annotated as a Fructose 1,6-bisphosphate aldolase (FBA, EC 4.1.2.13) protein among the genes screened. The FBA plays an important role in the third phase of the Calvin-Benson cycle, and its activity is regulated by redox post-translational modification (PTM) [57]. In alfalfa, MsCML10 decodes the cold-induced Ca^2+^ signal and regulates cold tolerance by activating MsGSTU8 and MsFBA6, which subsequently lead to enhanced maintenance of ROS homeostasis and elevated accumulation of sugars for osmoregulation, respectively [58]. It was found that the reducing sugar content and ROS scavenging capacity of TaFBA-A10 overexpression lines were higher than those of WT and FBA mutants under low temperature stress. Overexpression of *TaFBA-A10* could regulate the Calvin cycle and glycolysis rate by increasing FBA activity, thus enhancing the low-temperature tolerance of plants [59]. The evm. model.scaffold_39.426 is annotated as glycerol-3-phosphate acyltransferase (GPAT) is an important rate-limiting enzyme in triacylglycerol (TAG) biosynthesis, which is important for plant growth and development and abiotic stress response [60,61]. In rice, overexpression of the *AtS1* gene was reported to increase the unsaturation of fatty acids in phosphatidylglycerol (PG) and improve photosynthetic rate and growth at low temperatures [60,62]. Overexpression of the *AmGPAT* gene in *Ammopiptanthus mongolicus* improves tolerance to cold and other oxidative stresses in transgenic *A. thaliana* by increasing the level of unsaturated fatty acids in phosphatidylglycerol [63]. Meanwhile, we found that GPAT showed different degrees of up-regulated expression under low and rewarming treatments by expression profiling. The expression pattern in different tissue parts was observed to be highest in roots. Similarly, in barley, *HvGPAT14* also showed different expression levels in different tissues under low temperature [61].

Constructing a low-temperature-tolerant cDNA library of *D. odorifera* holds multifaceted scientific and application values, particularly in the study of plant stress biology and promoting sustainable forestry development. A cDNA library contains information about all mRNAs expressed under specific conditions. By constructing a cDNA library for *D. odorifera* under low-temperature conditions, we can identify genes that are activated or suppressed under cold stress, which will help elucidate how plants cope with cold adversity. Moreover, through screening the cDNA library, genes of interest can be isolated and functionally validated, playing a crucial role in assessing their contribution to enhanced cold tolerance. Additionally, low-temperatureresponsive genes identified can be utilized in genetic engineering technology, enabling the enhancement of low-temperature tolerance in *D. odorifera* and other crops via transgenic methods. This is significantly important for expanding cultivation areas and improving crop yield and quality.

## Supporting information

S1 Table Primers used in this study. (XLSX)

S2 Table FPKM values under cold treatment of candidate genes. (XLSX)

S3 Table FPKM values of different tissues of candidate genes. (XLSX)

S4 Table Sequencing results of candidate genes. (XLSX)

S5 Table Information of candidate genes. (XLSX)

## Abbreviations

BP: Biological process
CBF: Core binding factor
CC: Cellular component
FBA: Fructose 1,6-bisphosphate aldolase
GO: Gene Ontology
GPAT: Glycerol-3-phosphate acyltransferase
ICE: Inducer of CBF expression
KEGG: Kyoto Encyclopedia of Genes and Genomes
MDA: Malondialdehyde
MF: Molecular function
MT: Melatonin
MYC: Myelocytomatosis
NGS: Next-generation sequencing
PCR: Polymerase Chain Reaction
PG: Phosphatidylglycerol
POD: Peroxidase
qRT-PCR: Quantitative reverse transcription-PCR
SOD: Superoxide Dismutase
TAG: Triacylglycerol
TF: Transcription Factor.
